# Hygienic behaviour in Brazilian stingless bees

**DOI:** 10.1242/bio.018549

**Published:** 2016-10-17

**Authors:** Hasan Al Toufailia, Denise A. Alves, José M. S. Bento, Luis C. Marchini, Francis L. W. Ratnieks

**Affiliations:** 1 Laboratory of Apiculture and Social Insects, School of Life Sciences, University of Sussex, Brighton BN1 9QG, UK; 2 Departamento de Entomologia e Acarologia, Escola Superior de Agricultura “Luiz de Queiroz”, Universidade de São Paulo, Piracicaba, São Paulo 13418-900, Brazil

**Keywords:** Hygienic behaviour, Diseases, Stingless bees, *Melipona*, *Scaptotrigona*, *Tetragonisca*

## Abstract

Social insects have many defence mechanisms against pests and pathogens. One of these is hygienic behaviour, which has been studied in detail in the honey bee, *Apis mellifera*. Hygienic honey bee workers remove dead and diseased larvae and pupae from sealed brood cells, thereby reducing disease transfer within the colony. Stingless bees, Meliponini, also rear broods in sealed cells. We investigated hygienic behaviour in three species of Brazilian stingless bees (*Melipona scutellaris*, *Scaptotrigona depilis*, *Tetragonisca angustula*) in response to freeze-killed brood. All three species had high mean levels of freeze-killed brood removal after 48 h ∼99% in *M. scutellaris*, 80% in *S. depilis* and 62% in *T. angustula* (*N*=8 colonies per species; three trials per colony). These levels are greater than in unselected honey bee populations, ∼46%. In *S. depilis* there was also considerable intercolony variation, ranging from 27% to 100% removal after 2 days. Interestingly, in the *S. depilis* colony with the slowest removal of freeze-killed brood, 15% of the adult bees emerging from their cells had shrivelled wings indicating a disease or disorder, which is as yet unidentified. Although the gross symptoms resembled the effects of deformed wing virus in the honey bee, this virus was not detected in the samples. When brood comb from the diseased colony was introduced to the other *S. depilis* colonies, there was a significant negative correlation between freeze-killed brood removal and the emergence of deformed worker bees (*P*=0.001), and a positive correlation with the cleaning out of brood cells (*P*=0.0008). This shows that the more hygienic colonies were detecting and removing unhealthy brood prior to adult emergence. Our results indicate that hygienic behaviour may play an important role in colony health in stingless bees. The low levels of disease normally seen in stingless bees may be because they have effective mechanisms of disease management, not because they lack diseases.

## Abstract (precis)

**Summary:** Hygienic behaviour such as the removal of dead and diseased larvae and pupae has an important role in colony health in stingless bees, and it is greater than in unselected honey bee populations

## INTRODUCTION

Hygienic behaviour is one of a large number of defence mechanisms that honey bees, *Apis mellifera*, have against pests and diseases (Park, 1937; Rothenbuhler, 1964a,b; Spivak and Gilliam, 1998a,b; Wilson-Rich et al., 2009). In particular, it is a social defence against diseases of brood (larvae and pupae) in sealed cells. Worker honey bees showing hygienic behaviour detect and uncap cells containing dead and infected brood, and remove the contents from the colony (Rothenbuhler, 1964a,b). Hygienic behaviour has been shown to help control varroa mites, deformed wing virus (DWV), chalkbrood, and American foulbrood (Al Toufailia et al., 2014; Spivak and Gilliam, 1998a,b; Wilson-Rich et al., 2009). Hygienic behaviour is not learned, rather, it is an instinctive heritable trait controlled by multiple genetic loci (Jones and Rothenbuhler, 1964; Momot and Rothenbuhler, 1971; Rothenbuhler, 1964a,b; Wilson-Rich et al., 2009). Hygienic behaviour does not result in the excess removal of healthy brood (Bigio et al., 2014b) or reduce honey production (Spivak and Reuter, 1998b).

Historically, honey bee hygienic behaviour was investigated by introducing the spores of *Paenibacillus larvae*, the causative agent of American foulbrood, into cells with young larvae by killing whole frames of brood using hydrogen cyanide gas, by wounding pupae in capped cells with a pin inserted through the wax capping, by freezing whole brood frames, or by cutting out patches of brood from combs to be frozen and then placed back into the hive (Rothenbuhler, 1964a,b; Spivak and Downey, 1998). Nowadays, the level of hygienic behaviour in a colony is commonly determined by killing an area of capped cells by freezing with liquid nitrogen *in situ* (Spivak and Downey, 1998). Two days later, the cells with dead brood are checked to determine the proportion cleaned out. Unselected populations of honey bees show wide variation among colonies in the level of hygienic behaviour, but with a low mean and with few colonies that have high levels of hygienic behaviour (Pérez-Sato et al., 2009). By selective breeding it is possible to obtain colonies that are fully hygienic, defined as >95% removal of freeze-killed brood within 2 days (Bigio et al., 2014a; Ibrahim et al., 2007; Spivak and Reuter, 1998a,b).

Stingless bees, Meliponini, are a large group of eusocial bees found in the tropics worldwide and in the southern subtropics (Michener, 2000). They are important pollinators of wild plants and are increasingly being studied and used in Brazil and other countries for crop pollination (Heard, 1999; Slaa et al., 2006) and honey production (Cortopassi-Laurino et al., 2006). Like honey bees, stingless bees live in colonies with a queen and many workers, and rear brood individually in cells. In contrast to honey bees, in which food is given progressively to larvae in open cells, in stingless bees the brood cells are mass provisioned. Cells are filled with larval food regurgitated by worker bees soon after construction and just before oviposition by the queen, and sealed immediately after oviposition (Michener, 1974). Stingless bees are known for their varied nest defence against predators (Kerr and de Lello, 1962; Shackleton et al., 2015; van Zweden et al., 2011), but compared to honey bees, far less is known about their diseases and disease resistance. An important Brazilian book on beekeeping with stingless bees (Nogueira-Neto, 1997) has only the barest mention of diseases, in contrast to the considerable attention given to diseases of honey bees in beekeeping books. Indeed, there are numerous books just on honey bee pests and diseases or pathology (Bailey and Ball, 2013; Morse and Flottum, 1997; Morse and Richard, 1990). This may be because stingless bees have fewer pathogens and diseases, or it may be because they have highly effective ways of controlling diseases so that disease problems are rarely seen.

Hygienic behaviour has been studied in two species of stingless bees from Mexico, *Melipona beecheii* and *Scaptotrigona pectoralis* (Medina et al., 2009), using the freeze-killed brood bioassay. On average *M. beecheii* colonies took 4.4 days to remove all the frozen brood whereas *S. pectoralis* took significantly less, 2.2 days (*n*=8 colonies per species). Compared to unselected honey bees, these are high levels of hygienic behaviour. Indeed, all eight colonies of *S. pectoralis* would have been considered highly hygienic, >95% removal within 2 days, by honey bee standards. In Brazil, *Plebeia remota* has also been studied for hygienic behaviour using pin-killed brood, which resulted in 96.4% removal after 48 h (Nunes-Silva et al., 2009).

The aim of the current project was to gather further information on hygienic behaviour in stingless bees. We chose one species each of the two genera studied in Mexico (*M. scutellaris*, *S. depilis*) and one additional species (*Tetragonisca angustula*) to test using the freeze-killed brood bioassay. In addition, we also studied the effect of hygienic behaviour on the removal of *S. depilis* brood suffering from a novel and naturally occurring disease or disorder that we fortuitously observed.

## RESULTS

### Experiment 1: removal rates of freeze-killed brood

**Fig. 1.**
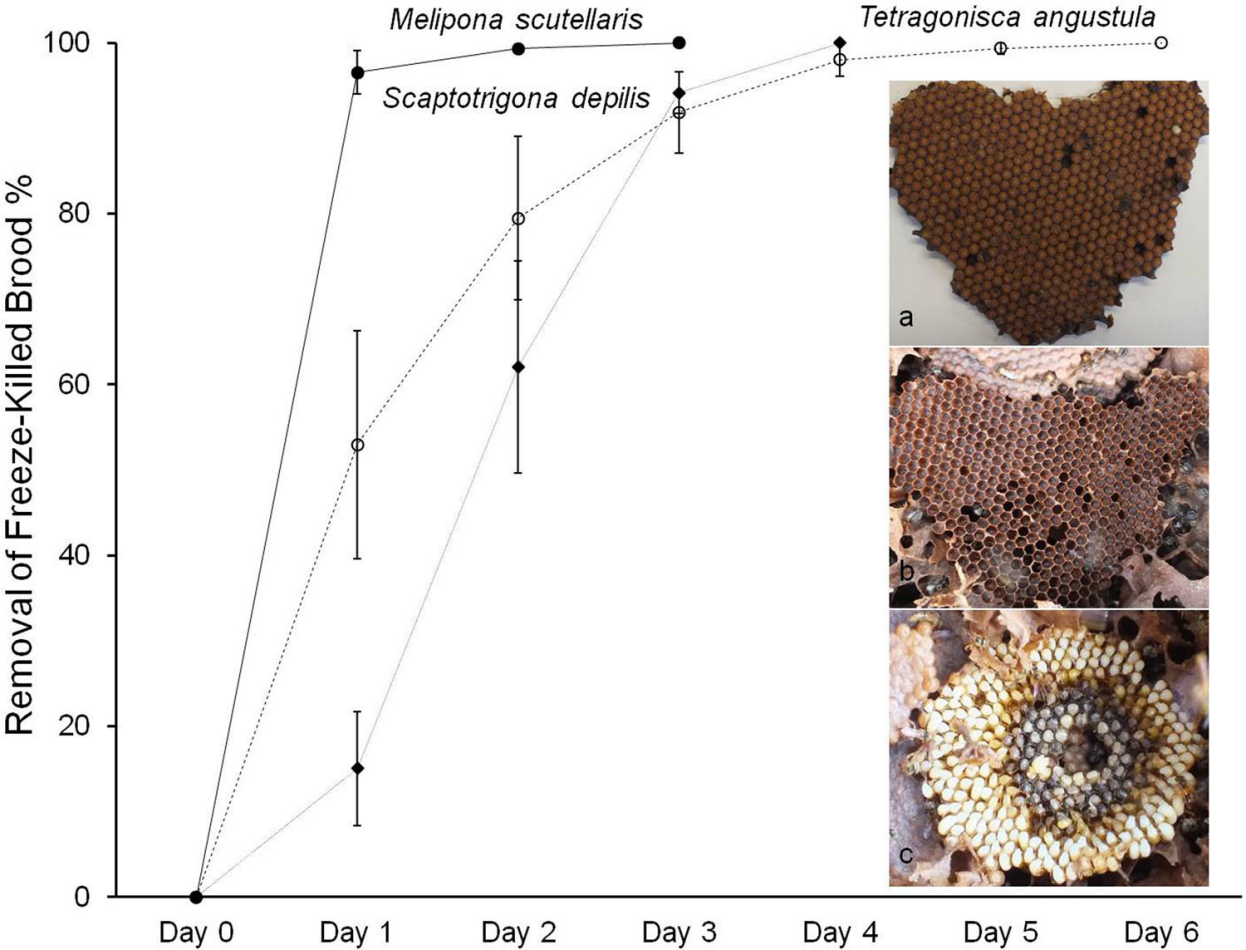
**Experiment 1: removal of freeze-killed brood in the three study species of stingless bees.** Each data point shows mean±standard error of 24 trials (eight colonies per species×three trials). (A) A piece of previously-frozen comb from *S. depilis* with ∼420 cells as placed into a test colony on day 0 of a trial; (B) the same piece of comb after 3 days with 100% of the dead brood removed; (C) a piece of previously-frozen comb after 1 day in a colony of *T. angustula* in which all cells have been uncapped but few dead brood have been removed.

There was no significant difference between the three trials (*F*=1.08; *P*=0.35). All three study species showed high levels of hygienic behaviour, in that freeze-killed brood were removed rapidly. After 2 days, the mean±s.e.m. removal of freeze-killed brood was 99.3±0.5% in *M. scutellaris*, 79.5±9.6% in *S. depilis* and 62±12.4% in *T. angustula* (Fig. 1). The time taken to remove all freeze-killed brood ranged from 1 to 3 days (1.3±0.2) in *M. scutellaris*, 2 to 4 days (3.0±0.3) in *T. angustula,* and 1 to 6 days (2.7±0.6) in *S. depilis* (Fig. 1). *M. scutellaris* was significantly faster than *S. depilis* and *T. angustula* (*P*<0.001) which were not significantly different from each other (*P*=0.58).

**Fig. 2.**
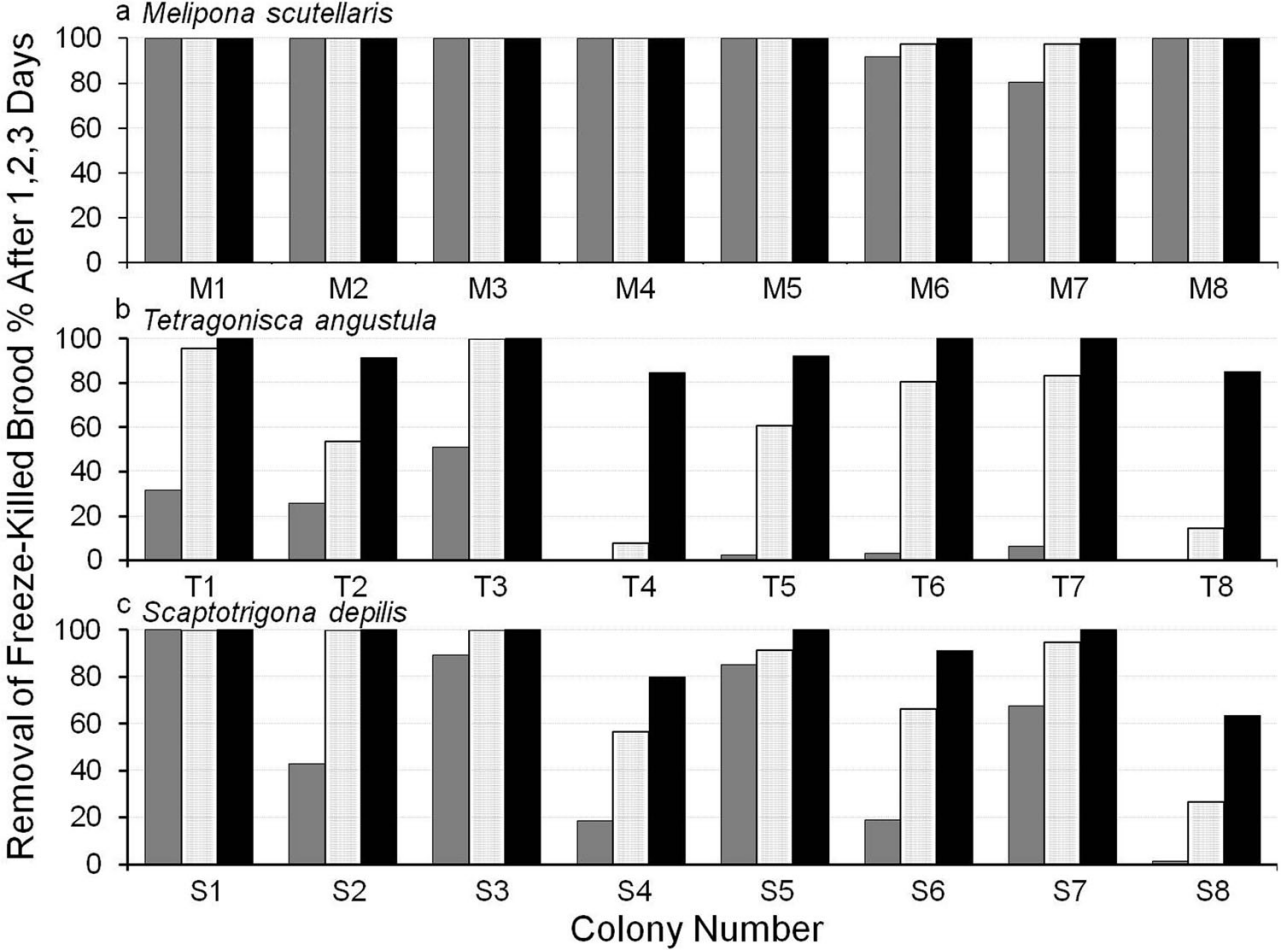
**Intercolony variation in hygienic behaviour, the removal of freeze-killed brood after 1, 2 and 3 days (dark grey, light grey, black, respectively), in *M. scutellaris*, *T. angustula* and *S. depilis*.** (A) In *M. scutellaris* there is very little intercolony variation; (B,C) in the other two species intercolony variation is much greater. Each histogram bar is the mean of three trials per colony.

Fig. 2 shows intraspecific variation in freeze-killed brood removal, which was significantly different between colonies within species (*F*=17.96; *P*<0.001). Variation is minimal in *M. scutellaris* but noticeable in the other two species, and especially in *S. depilis* which ranged between 27 and 100% removal after 2 days.

**Fig. 3.**
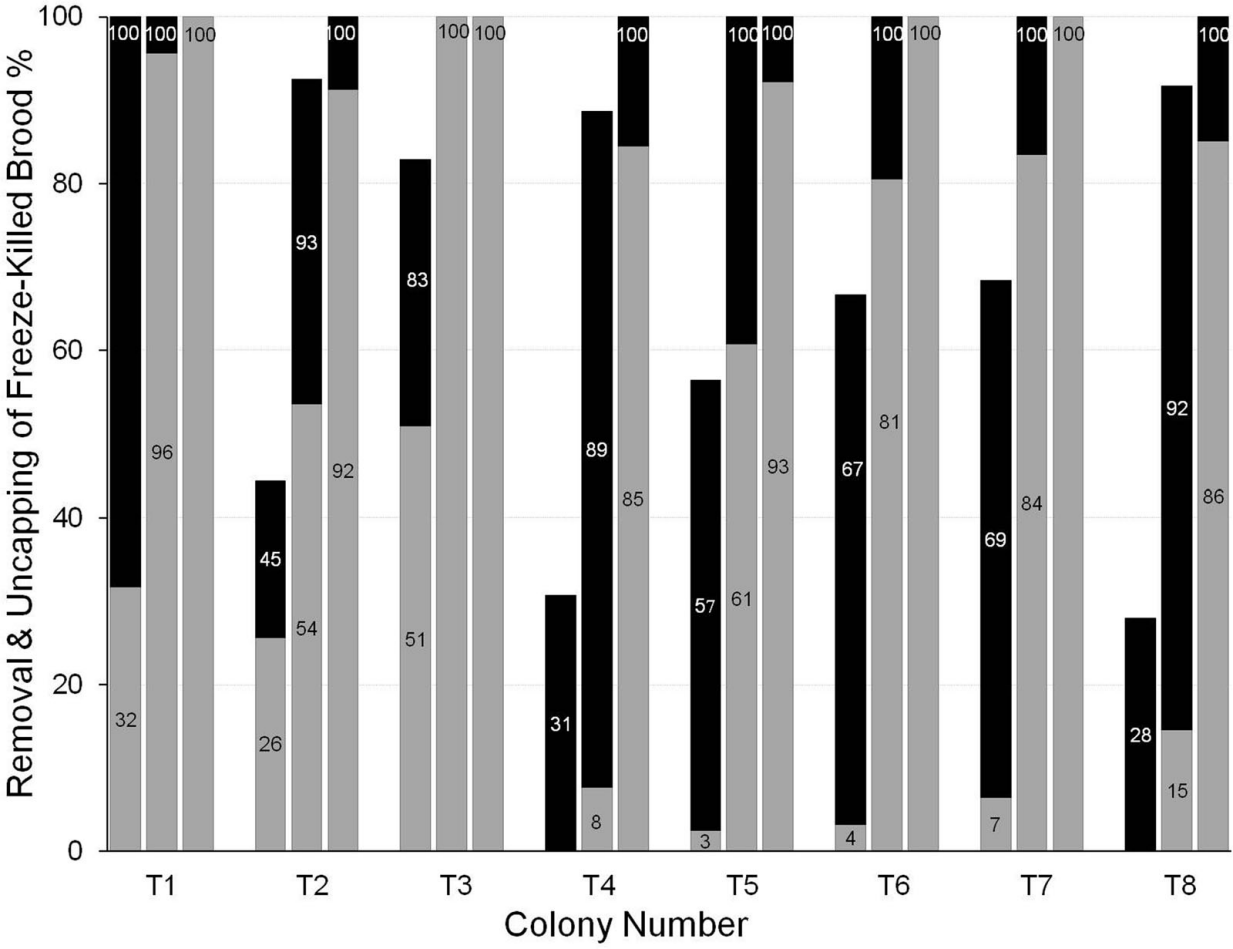
**Variation in uncapping and removing of freeze-killed brood between colonies of *T. angustula* 1, 2 and 3 days after introduction to the test colony.** Most of the variation between uncapping and removing was in the first day. Black: cells uncapped but dead brood not yet removed. Grey: cells contents removed.

In *T. angustula* the removal of freeze-killed brood in the first day was lower than in the other two species. In this species most colonies had a delay of approximately one day between uncapping a cell and then removing its contents (Fig. 3). 60±15.1% of the freeze-killed brood cells were uncapped in the first day (range: 28-100%) but only 15±10.4% were removed (range: 0-51%). By the end of the second day, 97±3.1% had been uncapped (range: 89-100%) and the contents had been removed from 62±13.9% (range: 8-100%). After 48 h colonies did not differ greatly in the proportion of uncapped cells, but differed more in the proportion from which the contents had been removed. For example, colonies 15 and 1 were able to uncap and remove the freeze-killed brood quicker than colonies 17 and 22.

### Experiment 2: removal of live brood in *S. depilis*, in which some cells produced workers with shrivelled wings

**Fig. 4.**
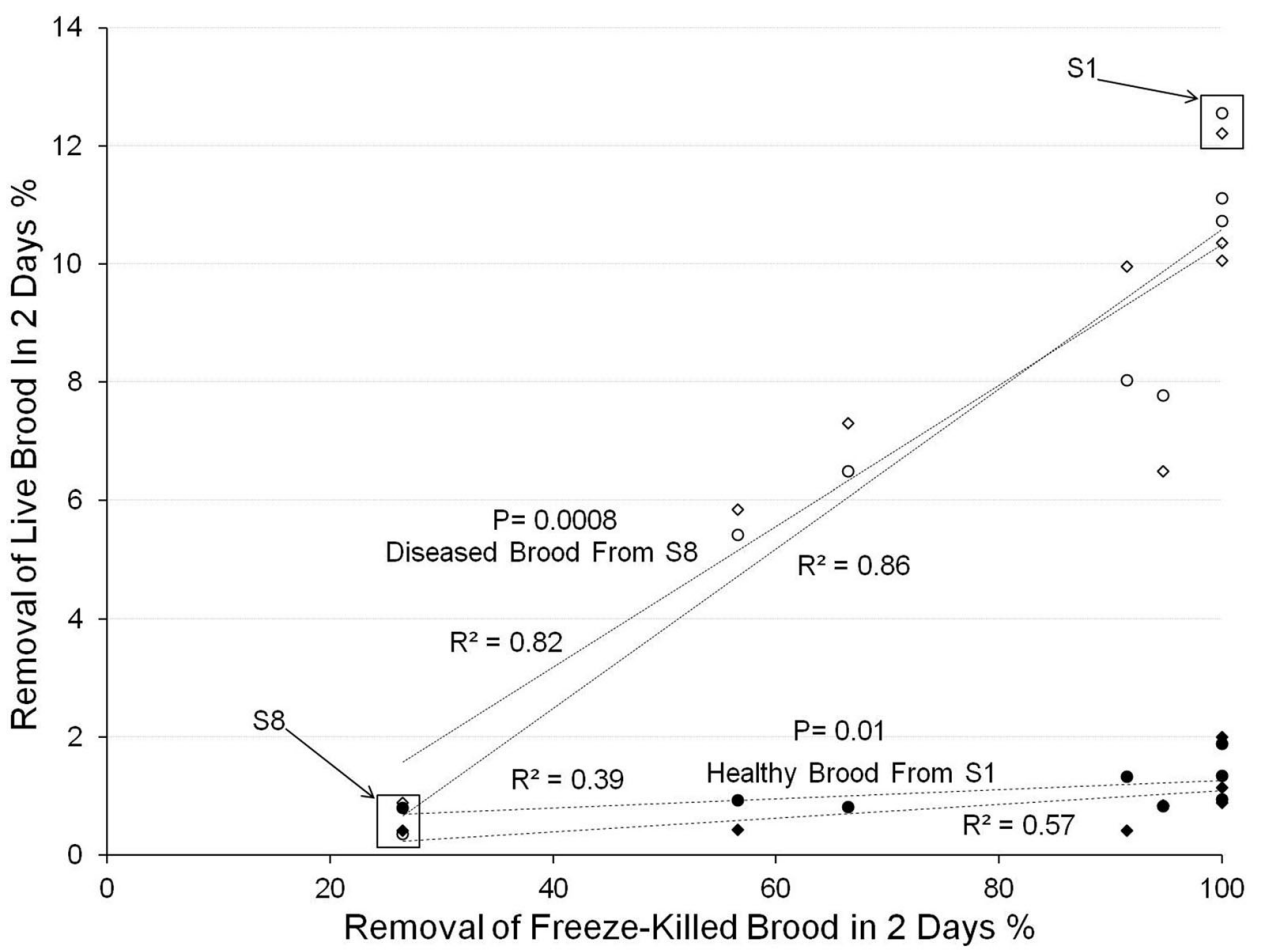
**Removal of live brood versus freeze-killed brood in eight colonies of *S. depilis*.** Colonies showing greater levels of hygienic behaviour against freeze-killed brood (Experiment 1) removed significantly more live brood taken from Colony S8, the Colony with unhealthy brood, but did not remove more healthy brood. The unhealthy brood all came from Colony S8 and the healthy brood from Colony S1. White data points refer to diseased brood from Colony S8. Black data points refer to healthy brood from Colony S1. Circles and diamonds refer to trials 1 or 2, respectively.

Fig. 4 shows that all eight *S. depilis* study colonies removed very few live healthy brood (1±0.2%) taken from Colony S1, showing a zero or negligible tendency to remove healthy non-nestmate brood (*F*=3.84; *P*=0.10). Removal of this healthy brood was not different between the two trials (*F*=2.31; *P*=0.15). However, colonies varied greatly in the removal of live unhealthy brood, taken from Colony S8 (0.5-12.5%). The proportion removed was positively and significantly correlated with the removal of freeze-killed brood in Experiment 1 (*F*=38.62; *P*=0.0008). Removal of live brood was not significantly different between the two trials (*F*=0.013; *P*=0.91) (Fig. 4).

**Fig. 5.**
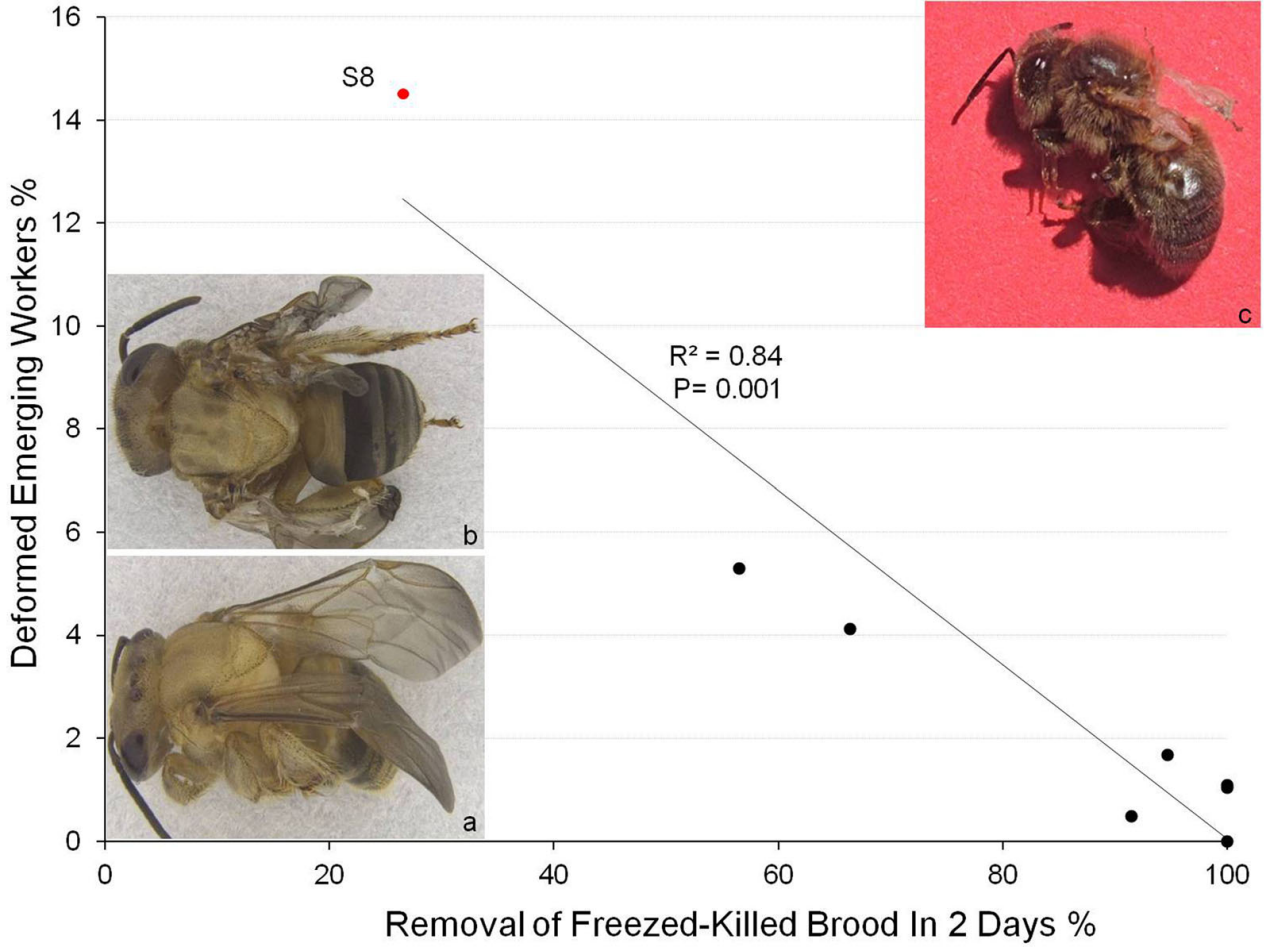
**Proportions of workers with shrivelled wings emerging from the combs taken from the *S. depilis* colony with unhealthy brood (Colony S8) in Experiment 2 versus freeze-killed brood removal (Experiment 1).** Significantly more unhealthy bees emerged from combs that had been kept for 5 days in less hygienic colonies. (A) A newly emerged worker *S. depilis* with normal wings; (B) a newly emerged worker *S. depilis* with shrivelled wings taken from Colony S8; (C) honey bee with shrivelled wings, as a result of deformed wing virus, for comparison. The red circle indicates the diseased colony of *S. depilis* (S8).

When the remaining live brood from Colony S8 were placed in an incubator to allow the adult bees to emerge from their cells, the opposite correlation was seen. That is, fewer workers with shrivelled wings emerged from brood combs taken from Colony S8 that had been placed in the colonies with greater removal of freeze-killed brood (*F*=32.4; *P*=0.001) (Fig. 5). This indicates that unhealthy brood had been removed via hygienic behaviour. The results of the two trials were very similar (*F*=0.03; *P*=0.86) (Fig. 4). The proportion of brood cells in Colony S8 giving rise to workers with shrivelled wings was ∼15%.

## DISCUSSION

The results of Experiment 1 show that the three stingless bee species studied all have high levels of hygienic behaviour, quantified as the removal of freeze-killed brood. Removal after 2 days was 99% in *Melipona scutellaris*, 80% in *Scaptotrigona depilis* and 62% in *Tetragonisca angustula* (*N*=8 colonies per species; three trials per colony). This is much greater than in unselected populations of the honey bee (46%; Pérez-Sato et al., 2009) and similar to previous research on the stingless bees *M. beecheii* and *S. pectoralis* in Mexico (Medina et al., 2009).

In *T. angustula* there was a delay of approximately one day between uncapping cells and removing the contents. In the honey bee, *A. mellifera*, Rothenbuhler's (1964b) classic experiment on behavioural genetics showed that the uncapping of cells containing diseased larvae and the removal of the diseased larvae from uncapped cells are distinct behaviours which are, at least partly, under the control of different genes. Further study will be needed to determine if this is the case in *T. angustula*. However, our results are also compatible with an alternative mechanism: that, for some unknown reason, there is simply a greater delay between uncapping and removing in this species than in the other study species.

Our results also showed considerable intercolony variation in the time taken to remove freeze-killed brood in *T. angustula* and, most noticeably (Fig. 2), in *S. depilis*. This is more similar to the situation in the honey bee, in which there is great variation among colonies in the proportion of freeze-killed brood removed within 2 days (Pérez-Sato et al., 2009). In *S. depilis*, intercolony variation in the removal of freeze-killed brood (Experiment 1, Fig. 1) was positively correlated with the removal of brood from combs that were naturally producing workers with shrivelled wings (Figs 4 and 5) (*P*=0.0008). This is important as it shows that the freeze-killed brood bioassay, in which brood are experimentally killed, was relevant to a naturally occurring brood disease.

The colony producing adult workers with shrivelled wings, Colony S8, removed only ∼27% of freeze-killed brood in Experiment 1, and only cleaned out ∼1% of live brood cells taken from Colony S8 when ∼15% of these contained a larva or pupa that would produce an adult with shrivelled wings if not removed (Fig. 5). By contrast, *S. depilis* colonies that had removed a greater proportion of freeze-killed brood in Experiment 1 cleaned out more cells from comb taken from Colony S8. This resulted in a smaller proportion of adults with shrivelled wings emerging from these cells. These data show clearly that the more hygienic colonies were removing brood that would otherwise have gone on to produce crippled adults, but that Colony S8 was removing none or almost none of these.

Experiment 2 also shows that more hygienic *S. depilis* colonies do not remove a greater number of healthy brood that non-hygienic colonies (Fig. 4). This has a parallel to recent research on hygienic behaviour in the honey bee, *A. mellifera*, which also found that colonies that removed higher proportions of freeze-killed brood did not remove higher proportions of healthy brood (Bigio et al., 2014b).

Overall, the results of this study and those of previous research (Nunes-Silva et al., 2009; Medina et al., 2009) suggest that most or all stingless bee species and most colonies exhibit high levels of hygienic behaviour, removing brood that has been killed by freezing, or in the case of *S. depilis*, by an as yet unidentified disease. The presence of one *S. depilis* colony, which was producing many workers with shrivelled wings and had a low level of hygienic behaviour, leads to the hypothesis that hygienic behaviour plays an important but previously unrecognized role in combating brood diseases in stingless bees. If this is the case, then the low levels of disease normally seen in stingless bees may be because they have effective mechanisms of disease management, and not simply because they do not have diseases. Our discovery of worker bees with shrivelled wings in *S. depilis* should be followed up in order to determine the cause, and in particular if is caused by a pathogen. Furthermore, our discovery of great intercolony variation in hygienic behaviour in *S. depilis* has implications for beekeeping with this species, and also in the further development of queen rearing and breeding methods now underway (Menezes et al., 2013). Selection could take place during queen rearing to produce colonies that show high levels of hygienic behaviour for commercial use. In addition, selection could take place to produce colonies with low levels of hygienic behaviour, for use in further research on the underlying mechanisms of hygienic behaviour and its importance in colony health and performance.

It is unclear why stingless bees have faster hygienic behaviour than unselected populations of the honey bee, *A.* *mellifera*. One possibility is that honey bees may have two alternative strategies against diseased larvae and pupae in sealed cells: either remove the diseased brood rapidly before spore formation or leave the brood permanently in the sealed cell (Spivak and Gilliam, 1991, 1993). Either strategy could reduce transmission of any pathogen. Rapid removal may reduce the infection of other brood within the colony as some brood diseases do not immediately become infective on killing the host larva. In the case of *Paenibacillus larvae*, the causative agent of American Foulbrood in *A. mellifera*, the bacteria must transform from rod to spore to become infective, so that a freshly killed larva is not infective (Ratnieks, 1992). It seems that different honey species may adopt different strategies; for example, *A*. *florea* removes the diseased brood rapidly (Woyke et al., 2012) and *A*. *cerana*, *A*. *dorsata* and *A*. *laboriosa* leave the brood permanently in the sealed cell (Boecking, 1999; Woyke et al., 2004). However, stingless bees never reuse cells (Nogueira-Neto et al., 1997). All brood cells are always torn down after being used and new cells are constructed. This is in contrast to honey bees, *Apis*, which reuse brood cells. Because stingless bees have to remove diseased brood from sealed cells anyway, it is probably advantageous to remove it rapidly. More generally, the reasons why individual colonies and species of bees that rear brood in sealed cells vary so much in the speed of hygienic behaviour is not well understood, and is an important general question for future research and hypotheses. Previous research has suggested that in *A. dorsata*, sealed cells with dead brood are not uncapped as this bee species is migratory and nests are short-lived, meaning that a few permanently sealed cells will not greatly reduce the availability of cells (Woyke et al., 2004). However, *A. cerana* also adopts the same strategy and is non-migratory (Boecking, 1999).

## MATERIALS AND METHODS

### Study site and species

The study was carried out in the Laboratory of Useful Insects (Laboratório de Insetos Úteis) at the University of São Paulo “ESALQ”, Piracicaba, São Paulo State, Brazil, between 21 February and 22 March 2015. The study colonies of *Scaptotrigona depilis* and *Tetragonisca angustula* were kept in wooden box hives [inside measurements 26×26×35 (high) cm and 25×13×13 (high) cm, respectively] in an outdoor meliponary shelter beside the laboratory. The wooden hives [inside measurements 43×24×18 (high) cm] of *Melipona scutellaris* were kept in a brick laboratory building with tubes leading from the hive entrances through holes in the walls to the outside to allow foraging. All colonies had a queen, brood of all ages, and pollen and honey stores, and were typical of their species in regard to colony size. Colonies were of similar population within a species, and the combs and food pots filled most but not all of each hive (mean: *M. scutellaris* 70% of hive filled, *S. depilis* 63%, *T. angustula* 80%).

The three study species were chosen as they are among the species of stingless bees most used in Brazil for honey production and pollination. For example, controlled queen-rearing methods are currently being developed for *S. depilis* in order to provide a supply of colonies for crop pollination (Menezes et al., 2013). As a result, information on disease resistance is of value, and could also be incorporated into a breeding program.

### Experiment 1: removal rates of freeze-killed brood

At the start of the study, each colony was inspected and a brood comb containing larvae and pupae was removed and divided into several pieces. Each piece was put into a plastic petri dish with lid and frozen at −20°C. These pieces were then used in a series of three trials of freeze-killed brood removal, one per week, starting 2 days later. Each trial used an average of 319.5±10.4 (mean±s.e.m.) sealed brood cells.

To carry out a trial, one or two pieces of the previously frozen brood comb were photographed to count the number of capped cells. These comb pieces were then placed into the top of each study hive (*n*=8 per species). The hive was then inspected every 24±1 h for the next 6 days or until all the previously frozen brood had been removed. Daily photographs were taken to determine the number of brood cells that still remained, and whether these had been fully cleaned out or just uncapped.

### Experiment 2: removal of live brood in *S. depilis*, in which some cells produced workers with shrivelled wings

At the start of the study we noticed that below one of our *S. depilis* study colonies (Colony S8) there were many young worker bees on the concrete floor of the meliponary shelter. On inspection, we saw that these had shrivelled wings (Fig. 5B). Experiment 1 showed that Colony S8 had the lowest level of freeze-killed brood removal of the eight study colonies of *S. depilis*. Therefore, we hypothesised that more hygienic *S. depilis* colonies would be able to detect and clean out cells with these diseased or disordered brood leading to lower production of adult worker bees with shrivelled wings.

To test this hypothesis we introduced patches of live brood (cells containing older larvae and pupae; mean 185±8.0 sealed cells), taken from Colony S8 into all eight *S. depilis* study colonies and quantified the cleaning out of cells for 5 days. We also introduced a patch of live brood taken from a healthy colony (Colony S1) into all eight colonies. This was a non-nestmate control, to allow for the possibility that removal was not due to disease but because the comb had come from another colony. We monitored the combs as before by taking daily photographs. In addition, at the end of the test period each piece of live comb was placed into an incubator (28°C), to allow the worker bees to emerge from their cells. We then determined the proportion emerging with shrivelled wings. We carried out two trials during the final 2 weeks of the study period.

The cause of the shrivelled-wing workers remains to be determined. Their overall appearance was similar to that of honey bee workers with overt symptoms of deformed wing virus (DWV) (Al Toufailia et al., 2014). However, cDNA generated from RNA extracted from pooled samples of workers from both the diseased colony and healthy *S. depilis* colonies did not amplify for primers of DWV-F2 (strain A) and DWV-R2a (strain B) (McMahon et al., 2015). Nevertheless, the unidentified cause was naturally occurring, and resulted in a serendipitous opportunity to gather important additional information of the role of hygienic behaviour in removing unhealthy brood from sealed cells.

### Statistical analysis

Data were analysed using the IBM SPSS statistical program version 20. If necessary we log- or arcsine-transformed the response variable to meet the assumptions of ANOVA (Grafen and Hails, 2002; Zuur et al., 2010). We then used ANOVA to test the effect of colony, trial and species on the time taken to remove all freeze-killed brood. Linear regression was used to test for the effects of hygienic behaviour on the removal of live brood from Colony S8. *P*<0.05 is defined as significant. Descriptive statistics are given as mean±standard error (s.e.m.).
